# Interaction among Skeletal Muscle Metabolic Energy Systems during Intense Exercise

**DOI:** 10.1155/2010/905612

**Published:** 2010-12-06

**Authors:** Julien S. Baker, Marie Clare McCormick, Robert A. Robergs

**Affiliations:** ^1^Health and Exercise Science Research Laboratory, School of Science, University of the West of Scotland, Hamilton Campus, Almada Street, Hamilton ML3 0JB, UK; ^2^School of Human Movement Studies, Charles Sturt University, Bathurst, NSW 2795, Australia

## Abstract

High-intensity exercise can result in up to a 1,000-fold increase in the rate of ATP demand compared to that at rest (Newsholme et al., 1983). To sustain muscle contraction, ATP needs to be regenerated at a rate complementary to ATP demand. Three energy systems function to replenish ATP in muscle: (1) Phosphagen, (2) Glycolytic, and (3) Mitochondrial Respiration. The three systems differ in the substrates used, products, maximal rate of ATP regeneration, capacity of ATP regeneration, and their associated contributions to fatigue. In this exercise context, fatigue is best defined as a decreasing force production during muscle contraction despite constant or increasing effort. The replenishment of ATP during intense exercise is the result of a coordinated metabolic response in which all energy systems contribute to different degrees based on an interaction between the intensity and duration of the exercise, and consequently the proportional contribution of the different skeletal muscle motor units. Such relative contributions also determine to a large extent the involvement of specific metabolic and central nervous system events that contribute to fatigue. The purpose of this paper is to provide a contemporary explanation of the muscle metabolic response to different exercise intensities and durations, with emphasis given to recent improvements in understanding and research methodology.

## 1. Introduction

Muscle contraction and, therefore, all exercise are dependent on the breakdown of adenosine triphosphate (ATP) and the concomitant release of free energy (1). This free energy release is coupled to the energy requirements of cell work, of which muscle contraction is just one example

(1) $$\text{ATP}\overset{\text{ATPase}}{\to }\text{ADP}+\text{Pi}+\text{energy}\to \text{muscle}\;\text{contraction}.$$

One would think that muscle, like all cells, would benefit from a large store of ATP from which to fuel cell work. However, this is not the case. The total quantity of ATP stored within the cells of the body is very small (approximately 8 mmol/kg wet weight of muscle). Thus, cells rely on other mechanisms to supply ATP to support cell work, which involves the store of energy in more complex molecules such as glycogen and triacylglycerols, and more importantly, having a sensitive control system to rapidly increase metabolism during times of energy (ATP) demand. Muscle tissue is unique in that it can vary its metabolic rate to a greater extent than any other tissue depending on the demands placed upon it [1]. The study of bioenergetics provides a rationale explanation for this scenario, where the concentrations of muscle ATP, ADP, AMP, and Pi during rest conditions are optimal for supporting free energy transfer to and from ATP.

**Figure 1:**
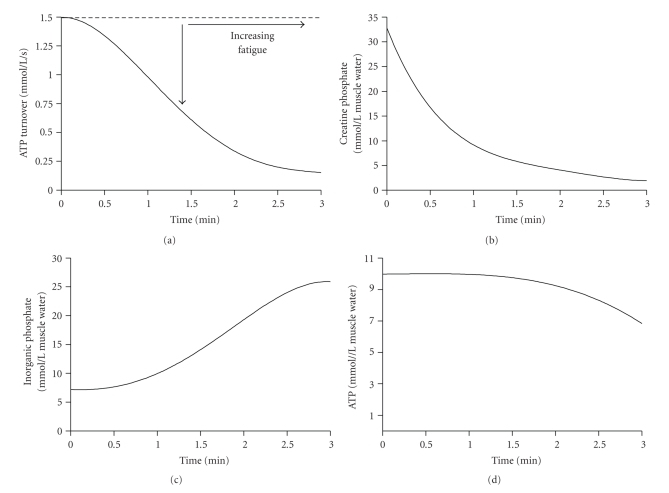
Stack plots of the change in (a) ATP turnover and key phosphagen system metabolites during 3 minutes of intense exercise to volitional exhaustion. (b) Creatine phosphate, (c) inorganic phosphate, and (d) ATP.  Note the well maintained preservation of muscle ATP for most of the duration of the exercise bout.

All cells function to maintain the resting condition adenylate metabolite concentrations as best as possible in the face of increasing ATP demand. The best example of this trait of cellular energy metabolism is the relatively stable muscle ATP concentration despite more than a 1,000-fold increase in ATP demand, which can occur during short-term intense exercise (Figure 1). For example, muscle ATP decreases by only 1 to 2 mmol/kg wet wt during these conditions, and even with involuntary maximal contraction to contractile failure, muscle ATP does not get lower than 5 mmol/kg wet wt [2]. In short, the muscle ATP concentration is not an energy store, but collectively with each of ADP, AMP, and Pi is an essential requirement for optimal cell function. Furthermore, any reduction in muscle ATP coincides with cellular conditions associated with the rapid development of fatigue, defined as a reduction in the ability of a muscle to produce force or power, or a reduction in ATP turnover of skeletal muscle [3, 4]. Fatigue is vital to the physiological function of the human body as it prevents ATP falling to such low levels that could cause muscle rigor or irreversible muscle damage [5–9].

**Figure 2:**
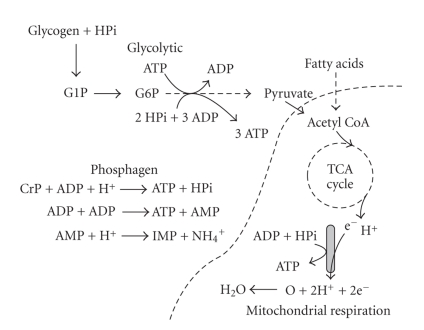
The three energy systems of muscle ATP regeneration.

How then can cells detect, rapidly respond to, and successfully meet sudden increases in ATP demand? The answers lie in an understanding of the systems by which cells regenerate ATP. There are three major energy systems which are responsible for the resynthesis of ATP (Figure 2). These systems can be categorised as follows: (1) The Phosphagen System, (2) The Glycolytic System, and (3) Mitochondrial Respiration.

The purpose of this paper is to re-explain the simultaneous and coordinated contributions of all energy systems to meet muscle ATP demand during different intensities and durations of exercise. It is important to provide a contemporary perspective of muscle metabolism given recent advances in understanding of energy system interaction, novel findings from advanced technologies such as magnetic resonance spectroscopy (MRS), and consensus from current debates on the biochemistry and cellular implications of metabolic acidosis.

## 2. The Phosphagen System

There are three reactions that comprise the phosphagen system, and these are presented in (2), and illustratively in Figure 2

(2) $$\begin{array}{c} \text{CrP}+\text{ADP}+{\text{H}}^{+}\overset{\text{creatine}\;\text{kinase}}{\to }\text{ATP}+\text{Cr},\\ \text{ADP}+\text{ADP}\overset{\text{adenylate}\;\text{kinase}}{\to }\text{ATP}+\text{AMP},\\ \text{AMP}+{\text{H}}^{+}\overset{\text{AMP}\;\text{deaminase}}{\to }\text{IMP}+{\text{N}{\text{H}}_{4}}^{+}. \end{array}$$

The creatine kinase and adenylate kinase reactions both produce ATP, yet the creatine kinase reaction has by far the greater capacity for ATP regeneration as the store of CrP in muscle at rest is approximately 26 mmol/kg wet wt. The proton (H^+^) consumption during the creatine kinase reaction accounts for the slight alkalinization of muscle at the onset of exercise. The onset of metabolic acidosis activates AMP deaminase and therefore the production of AMP and eventually the production of ammonia (NH_4_^+^). The small capacity of this reaction in skeletal muscle in combination with the pre-existence of acidosis makes the H^+^ consumption of this reaction of limited consequence.

The other important feature of the phosphagen system, and in particular the adenylate kinase reaction, is the production of AMP. AMP is a potent allosteric activator of two enzymes influential to glycolysis. First, AMP activates phosphorylase, which increases glycogenolysis and therefore the rate of glucose-6-phosphate (G_6_P) production, which in turn provides immediate fuel for glycolysis. Second, AMP activates phosphofructokinase (PFK) within phase 1 of glycolysis, thereby allowing increased flux of G_6_P through glycolysis, which in turn allows for increased rates of ATP regeneration from phase 2.

The third reaction, the AMP deaminase reaction, does not regenerate ATP. Nevertheless, we like to include this reaction within the phosphagen system as theoretical understanding of bioenergetics reveals that converting AMP to IMP is necessary to aid in the retention of a higher than otherwise phosphate transfer potential within muscle [10]. In other words, keeping AMP and ADP low within muscle, despite small reductions in ATP, can sustain sufficient free energy release during ATP hydrolysis to provide adequate energy to fuel muscle contraction. Approximately 1-2% of the Caucasian population are believed to have a skeletal muscle AMP deaminase deficiency [11–13]. Studies have suggested that these individuals are more likely to suffer from exercise-induced cramping, pain, and early fatigue [12, 14]. 

**Figure 3:**
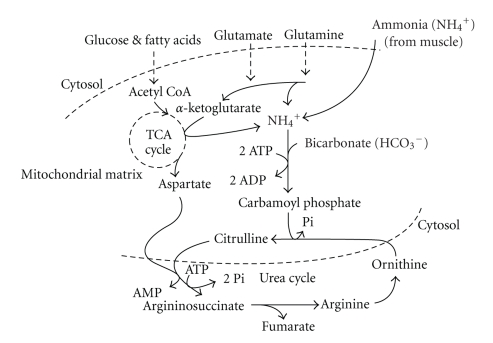
The urea cycle of the liver, showing the connections between the TCA cycle in the mitochondria, ammonia, and the production of urea. Note that the conversion of glutamate to *α*-ketoglutarate (top central region of figure) is condensed for clarity (oxaloacetate + glutamate → *α*-ketoglutarate + aspartate [*aspartate aminotransferase*]) and is therefore also a source of aspartate. The direct conversion of glutamate to *α*-ketoglutarate is catalyzed by *glutamate dehydrogenase*.

**Figure 4:**
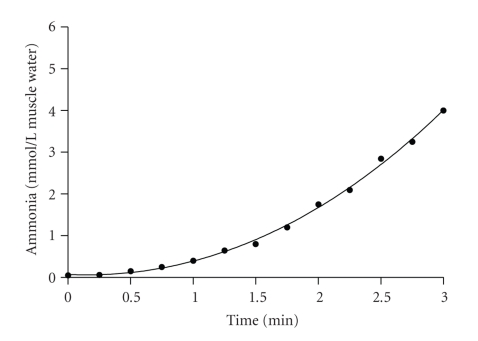
Data for the increase in muscle ammonia during 3 minutes of intense exercise to fatigue. Data are modelled from known changes in muscle IMP and amino acid oxidation. Adapted from Spriet et al. [2], (1987a, 1987b), Medbo et al. [18], Kemp et al. [19], and Bendahan et al. (2003).

The other important feature of the AMP deaminase reaction is the production of ammonia (NH_4_^+^), which is toxic to cells and subsequently removed into the blood for circulation to the liver and subsequent conversion to urea, this process is known as the urea cycle (Figure 3). Although this reaction is not the only source of ammonia during intense exercise, as some is also produced from amino acid oxidation, it accounts for most ammonia production, which as shown in Figure 4 can be quite substantial during sustained intense exercise to fatigue [15]. Nevertheless, blood ammonia does not increase to high levels, with peak concentrations during incremental exercise approximating 0.1 mmol/L [16]. Nevertheless defects in the urea cycle can occur which cause elevated levels of ammonia in the blood which can ultimately lead to irreversible brain damage [17]. Given the importance of muscle and whole body amine group balance in topics pertaining to exercise and muscle protein balance in athletes and the elderly alike, understanding the fate of amine groups during energy catabolism will become a more important topic within exercise biochemistry and physiology in the future.

**Figure 5:**
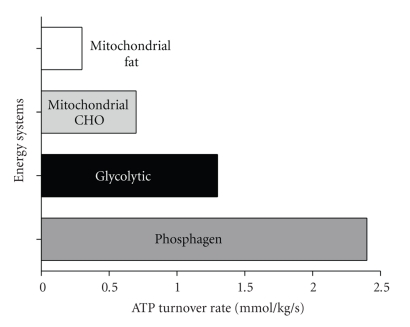
Maximal rates of ATP regeneration from the energy systems of skeletal muscle. Adapted from Sahlin et al. [25].

Many activities have a high dependence on the phosphagen system. Success in team sports, weight lifting, field events (e.g., shot put and discus throwing, jumping events), swimming, tennis, and so forth. All require short-term singular or a limited number of repeated intense muscle contractions. It has long been theorized that during the initial 10–15 seconds of exercise that creatine phosphate was solely responsible for ATP regeneration [6]. Added support for the theory of a near sole dependence on creatine phosphate during intense exercise arose because creatine phosphate is stored in the cytosol in close proximity to the sites of energy utilisation. Phosphocreatine hydrolysis does not depend on oxygen availability, or necessitate the completion of several metabolic reactions before energy is liberated to fuel ATP regeneration. However, as will be discussed in the section on glycolysis, a growing body of research has shown that glycolysis is rapidly activated during intense exercise, and seldom is there near complete reliance on the phosphagen system [20]. Nevertheless, the importance of phosphagen system lies in the extremely rapid rates at which it can regenerate ATP, as shown in Figure 5. Although controversy exists between physiologists over the measurements of the components of the energy systems, namely, the power, capacity, and relative contribution of each system during exercise, it has been generally accepted that with an exercise period of maximal effort of up to 5 to 6 seconds duration, the phosphagen energy system dominates in terms of the rate and proportion of total ATP regeneration [21–23]. Evidence suggests that when high-intensity contractions commence, the rate of CrP degradation is at its maximum but begins to decline within 1.3 s [24].

During severe exercise the energy yield from the phosphagen system may continue until the stores of CrP are largely depleted (see Figure 8) [6, 26]. This can occur within 10 s of the onset of maximal exercise due to the exponential path of decay that CrP degradation has been found to follow [27]. Thus the energetic capacity of this system is dependent on the concentration of creatine phosphate.

**Figure 6:**
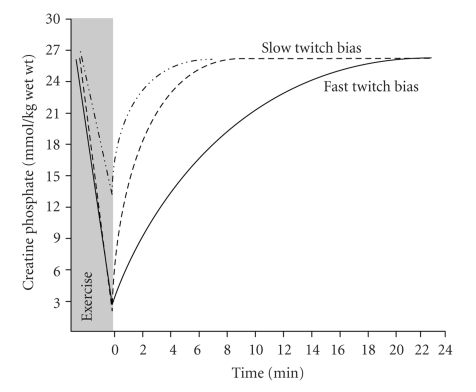
Representative kinetics of creatine phosphate (CrP) recovery in subjects with different end exercise CrP concentrations and different proportions (bias) of slow or fast twitch muscle. Note the more rapid recovery of CrP when there is less exercise-induced depletion (-*··*-*··*) versus near complete depletion (- - - -). Based on unpublished research observations of the authors.

Interestingly, most sports involve repeated bouts of intense exercise, separated by either active or passive recovery. Clearly, the rate of creatine phosphate recovery kinetics is also important to appreciate and understand the role of the phosphagen system in sports and athletics. The ability of athletes to repeatedly recover their CrP stores and therefore produce high power outputs can have a significant effect on the outcome of their performance. Research has shown that after exhaustive exercise, near complete replenishment of the creatine phosphate may take from <5 minutes to in excess of 15 minutes, depending on the extent of CrP depletion, severity of metabolic acidosis (slower if more acidic), and the muscle motor unit and fiber type characteristics of the exercised muscle. Such different rates of CrP recovery are presented in Figure 6, and data is based on our recent and as yet unpublished observations using phosphorous magnetic resonance spectroscopy (^31^P MRS). Unfortunately, limited research has been done to understand the implications of different rates of CrP recovery, or different strategies to improve such recovery [28].

**Figure 7:**
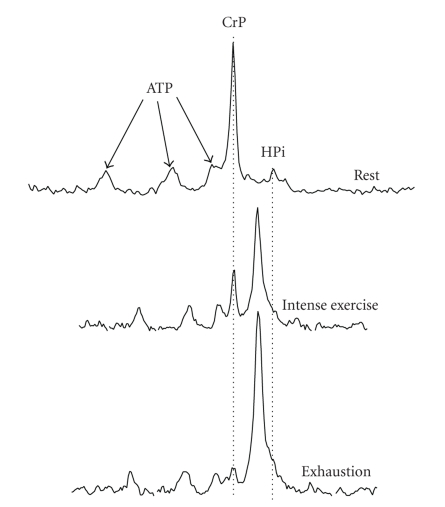
The ^31^P MRS spectrum of resting muscle (top), muscle at near complete CrP depletion (middle), and muscle with complete CrP depletion (bottom). Note the decrease in CrP and increase in HPi as well as the resonance frequency shift of the HPi peak relative to CrP, indicating acidosis. The muscle pH of each condition is 7.01, 6.74, and 6.18, respectively. Based on unpublished research observations of the authors.

It is important to understand the research methodology of ^31^P MRS, as since its introduction in the 1980s it has become the main method used to study the phosphagen system during and in recovery from exercise. Many research journals have also stated specific intentions to invite and publish more research based on ^31^P MRS methodology. Research using ^31^P MRS requires the use of a large bore magnet within which is a peripheral coil that is electronically tuned to the atomic signal frequency of the atom of interest. For example, when placed in a magnetic field, most atoms with a negative number of electrons will be forced to alter their alignment when subjected to a short burst of high-frequency energy. Once the pulse of energy is over, the atoms release their specific frequency of energy for the given magnetic field as they return to their stable state. This data collection occurs over several milliseconds, and the resulting data is referred to as a free induction decay (FID). It is this signal that is collected in all forms of magnetic resonance imaging and spectroscopy. For spectroscopy, the FID is mathematically processed by a procedure known as Fourier transformation, which essentially converts the data from numbers expressed over time, to numbers expressed relative to the frequency of change of the data. This processing produces a spectrum, where the curves, or peaks, represent the relative abundance of specific frequencies of change (Figure 7). For ^31^P MRS, the larger the area under these curves, the greater the concentration of the phosphorous containing metabolite to which they represent.

The ^31^P MRS spectrum for muscle at rest is shown in Figure 7. There are 5 signal peaks typically resolved, depending on the strength of the magnetic field and the extent of sample collection and averaging. The higher the magnetic field the stronger the signal and the higher the frequency of this signal for any given metabolite. Due to the magnetic field strength specificity of the signal frequency for a given atom, this frequency is corrected for the field strength, resulting in the common ppm *x*-axis unit of the MRS spectrum. This allows data from different magnets to be compared to one another.

Note that the frequency of signal for each phosphorous atom is slightly different for different molecules due to the influence of the local atomic environment of the phosphorous atom. Hence, the signal from the phosphorous of ATP is slightly different for each of the three phosphorous atoms of the three phosphate groups, which is different again from CrP, and different again from free inorganic phosphate (Pi). There is no peak for ADP or AMP, as the concentrations of these adenylates are far too low to be detected by ^31^P MRS. The area under the curve of each peak is proportional to metabolite concentration, and typically for human subjects research, the absolute concentration of each metabolite is computed based on an assumed internal reference standard for ATP of approximately 8 mmol/kg wet wt. The central, or *α*, ATP peak is used for this reference standard.

As well as the rate of CrP recovery it is also important to consider the nature of the recovery process. Evidence from previous studies which have looked at the nature of CrP resynthesis points towards CrP resynthesis having a biphasic recovery pattern following intense muscular contraction [10, 29]. It seems that there is an initial fast phase immediately after exercise followed by a slower secondary recovery phase. Harris et al. [30] used muscle biopsy of the quadriceps to study the nature of CrP resynthesis and found that following intense dynamic exercise the half time (*t*_1/2_) of the fast and slow components of CrP resynthesis was 21 and >170 s, respectively. They concluded if a monoexponential model was used to estimate CrP resynthesis then *t*_1/2_ would lie somewhere between the values for the fast and slow component. The final value for *t*_1/2_ would therefore depend on how long data was collected in the postexercise period. Also in support of the biphasic recovery of CrP, Bogdanis et al. [5] found that following a 30 seconds sprint on a cycle ergometer CrP was depleted to 19.5 ± 1.2% of resting levels immediately following the cessation of exercise. After 1.5 minutes of recovery CrP was restored to 65.0 ± 2.8% however after another 4.5 minutes of recovery CrP had only slightly increased further to 85.5 ± 3.5%. Mathematical models predicted that CrP resynthesis would not reach even 95% of resting value until 13.6 minutes after exercise. More recently Forbes et al. [31] studied CrP recovery kinetics in humans and in rats using ^31^P MRS. They found that in the majority of humans there was indeed an initial fast recovery component in skeletal muscle following intense exercise.

Nevertheless the evidence for the biphasic nature of CrP recovery is not conclusive. Although it seems unlikely that after intense exercise the model of recovery will follow a monoexponential pattern, it could be that a biphasic model may not be adequate to describe the resynthesis pattern. Advances in technology (e.g., ^31^P MRS) have shown that even in the first 3s of recovery the slope was significantly different to the slope in the first 0.5 s (*P* = .001) [32]. This suggests that there could be more than 2 distinct phases in the CrP recovery process.

There is conflicting evidence in the literature over the importance of oxygen during the resynthesis of CrP following high-intensity exercise. A number of studies have looked at recovery of muscle following high intensity exercise under ischaemic conditions. Sahlin and colleagues [25] and Harris and his coworkers [30] have found these conditions to substantially suppress the resynthesis of CrP. This therefore suggests that CrP resynthesis is reliant on oxidative metabolism [30, 33, 34]. However, Crowther and colleagues [35] found that following high-intensity exercise under ischaemic conditions, glycolytic flux remained elevated for a short period of time; it remained high for 3 seconds and had decreased to baseline levels within 20 seconds. If this was the case and glycolytic ATP production was making a considerable contribution to energy supply during the recovery phase, then an initial fast phase of CrP recovery would be expected immediately following the cessation of high-intensity exercise. This ties in with the work discussed previously. Work done by Forbes et al. [31] also suggests that glycolytic ATP production may have contributed the CrP resynthesis during the initial fast phase of recovery following high-intensity exercise. If CrP is only partially restored during the recovery phase, this can lead to a compromised performance in subsequent exercise bouts, for example, a decrease in power output.

## 3. Glycolysis

**Figure 8:**
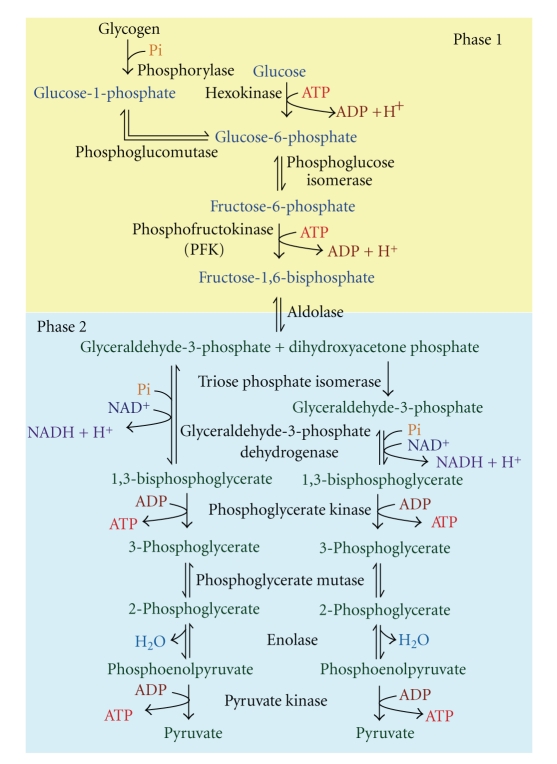
The glycolytic pathway. Note the duplication of phase 2 3-carbon metabolite reactions to account for the 6 carbons of each metabolite from phase 1.

When exercise continues longer than for a few seconds, the energy to regenerate ATP is increasingly derived from blood glucose and muscle glycogen stores [36]. This near immediate activation of carbohydrate oxidation after the onset of exercise [37] is caused by the production of AMP, the increases in intramuscular free calcium and inorganic phosphate (both increase the rate of the phosphorylase reaction as calcium is an activator of phosphorylate and inorganic phosphate is a substrate), and the near spontaneous increase in blood glucose uptake into muscle caused by muscle contraction. The increased rate of glucose-6-phosphate (G_6_P) production from glycogenolysis and increased glucose uptake provides a rapid source of fuel for a sequence of 8 additional reactions that degrades G_6_P to pyruvate. This sequence of reactions, or pathway, is called glycolysis (Figure 8). 

Glycolysis involves several more reactions than any component of the phosphagen system, slightly decreasing the maximal rate of ATP regeneration (Figure 5). Nevertheless, glycolysis remains a very rapid means to regenerate ATP compared with mitochondrial respiration [22]. It is convenient to separate glycolysis into two phases. Phase 1 involves six carbon phosphorylated carbohydrate intermediates called hexose phosphates. Phase 1 is also ATP costly, with ATP providing the terminal phosphate in each of the hexokinase and phosphofrucktokinase reactions. Phase 1 is best interpreted to prepare for phase 2, where ATP regeneration occurs at a higher capacity than the cost of phase 1, resulting in net glycolytic ATP yield.

Phase 2 is the ATP regenerating phase of glycolysis. Each reaction of phase 2 is also repeated twice for a given rate of substrate flux through phase 1, as phase 2 involves 3 carbon phosphorylated intermediates, or triose phosphates. Such a doubling of reactions is caused by the splitting of fructose-1,6-bisphosphate into dihydroxyacetone phosphate and glyceraldehyde-3-phosphate. Triosephosphate isomerase catalyses the conversion of dihydroxyacetone phosphate to glyceraldehyde-3-phosphate. Consequently, 2 molecules of glyceraldehyde-3-phosphate are now available for phase 2 of glycolysis, thereby allowing the doubling of each subsequent reaction when accounting for substrate flux and total carbons.

It is important to note the role of inorganic phosphate as a substrate in the glyceraldehyde-3-phosphate dehydrogenase reaction. This is a very exergonic reaction, allowing free inorganic phosphate to bind to glyceraldehyde-3-phosphate, forming 1,3-bisphosphoglycerate. It is this reaction that effectively allows for glycolysis to be net ATP regenerating as it provides the added phosphate group necessary to support additional phosphate transfer to ADP to form ATP in subsequent reactions. The two reactions that regenerate ATP in glycolysis are the phosphoglycerate kinase and pyruvate kinase reactions, resulting in 4 ATP from phase 2.

Close inspection of Figure 8 reveals that there is an added immediate ATP benefit from commencing glycolysis from glycogen versus glucose. We state “immediate” here because glucose needs to be phosphorylated to G_6_P prior to conversion to G_1_P and glycogen synthesis. However, this is normally done in the resting and postprandial state well before exercise commences. Once exercise and glycogenolysis begin, this earlier cost is benefited from and thereby increases net ATP regeneration from glycolysis from 2 to 3 ATP per G_6_P conversion to 2 pyruvate, which is a meaningful 50% increase in the rate and capacity of glycolytic ATP turnover.

**Figure 9:**
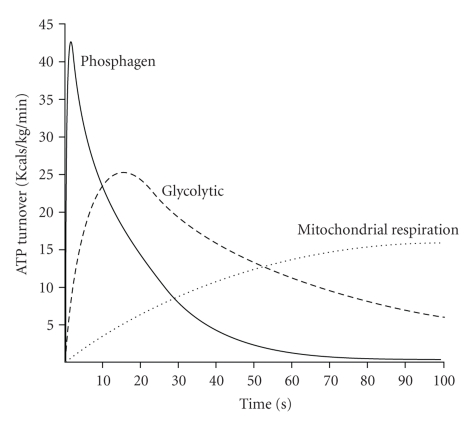
Energy system interaction and the differences in rates of ATP turnover during short term intense exercise to fatigue. The data presented is original, theoretical, and based on the authors' assessment of contemporary research evidence.

Traditionally within exercise science it was thought that CrP was the sole fuel used at the initiation of contraction, with glycogenolysis occurring at the onset of CrP depletion. However, we have learned from a variety of research studies that ATP resynthesis from glycolysis during 30 seconds of maximal exercise begins to occur almost immediately at the onset of performance [20, 38]. Also, unlike CrP hydrolysis which has a near instantaneous maximal rate of catalysis, ATP production from glycolysis does not reach its maximal rate of regeneration until after about 10 to 15 seconds of exercise and is maintained at a high rate for several more seconds. Over a period of 30-second of exercise the contribution from glycolysis to ATP turnover is nearly double that of CrP [39–42] (Figure 9). It has been estimated that during a 30 seconds sprint the phosphagen system accounts for 23% of energy provision, 49% comes from glycolysis and 28% from mitochondrial respiration. Whereas during a 10-second maximal sprint it has been estimated that energy is provided by 53% phosphagen, 44% glycolysis, and 3% mitochondrial respiration [43].

Maximum ATP regeneration capacity from glycolysis is achieved when a rate of work requiring an energy load greater than an individual's maximum oxygen uptake ($\dot{\text{V}}{\text{O}}_{2\;\max}$) is performed for as long as possible, which for the average trained athlete is between 2 to 3 minutes [44].

## 4. The Importance of Lactate Production

**Figure 10:**
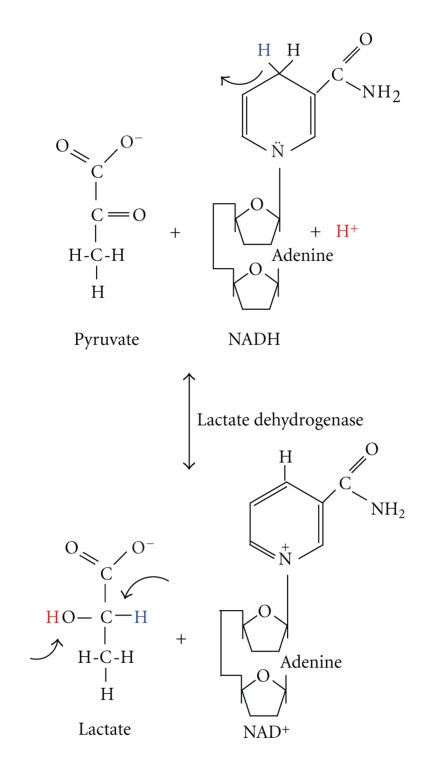
The lactate dehydrogenase reaction. Note the use of the proton to complete the reduction of pyruvate to lactate. Furthermore, the metabolic proton buffering provided by lactate production is largely pH independent.

Carbohydrate is the only nutrient whose stored energy can be used to generate ATP via glycolysis. When carbohydrate in the form of glucose or glycogen is catabolised during high-intensity performance only a partial breakdown or oxidation occurs, compared to the complete oxidation when reliant on mitochondrial respiration [46]. This is because pyruvate production occurs at rates that exceed the capacity of the mitochondria to take up pyruvate. To prevent product inhibition of glycolysis and a reduction in the rate of glycolytic ATP regeneration, as much pyruvate as possible must be removed from the cytosol. While some pyruvate is transported out of contracting muscle fibers, most is converted to lactate via the lactate dehydrogenase reaction (see (3) and Figure 10). During a 100 m sprint blood lactate levels can rise from 1.6 to 8.3 mM [17]

(3) $$\text{Pyruvate}+\text{NADH}+{\text{H}}^{+}\overset{\text{lactate}\;\;\text{dehydrogenase}}{\to }\text{Lactate}+\text{NA}{\text{D}}^{+}.$$

The production of lactate during exercise was discovered in the early 19th century by Berzelitus, who found the muscles of hunted stags to have elevated levels of lactic acid [47]. However it was not until the beginning of the 20th century that the biochemistry of energy metabolism began to be better understood [48]. This led to a number of studies which indicated that lactate was a dead-end waste product of glycolysis [49, 50] and a major cause of muscle fatigue [51]. However around the 1970s this view began to be challenged [52], and it has now been shown that lactate is in fact beneficial during intense exercise [45, 53]. Production of lactate in muscle during intense exercise is beneficial for removing pyruvate, sustaining a high-rate of glycolysis, and regenerating cytosolic NAD^+^, which is the substrate of the glyceraldehyde-3-phosphate dehydrogenase reaction (Figure 8). This reaction, in being a dehydrogenase reaction, is also an oxidation:reduction reaction. Two electrons and one proton are removed from glyceraldehyde-3-phosphate and used to reduce NAD^+^ to NADH. Without enough NAD^+^ availability in the cytosol, the rate of this reaction would slow drastically, thereby constraining the rate of ATP regeneration of glycolysis. Herein is a tremendously important function of lactate production.

An added benefit of lactate production concerns the metabolic proton buffering. The lactate dehydrogenase reaction uses two electrons and one proton from NADH and a second proton from solution to reduce pyruvate to lactate. As such, lactate production retards, not causes, the development of metabolic acidosis.

In summary, muscle production of lactate is essential to remove pyruvate, regenerate NAD^+^ to sustain a high rate of ATP regeneration from glycolysis, and contribute to metabolic proton buffering. It is fair to state that we could not sustain high-intensity exercise for much longer than 10 to 15 seconds without lactate production.

## 5. Glycolysis and Lactate Production

Given the need for lactate production to provide sufficient NAD^+^ to support sustained high substrate flux through glycolysis, it is beneficial to combine glycolysis and lactate to assess the balance of net substrates and products for the glycolytic system. As will be shown, this presentation is also beneficial for revealing the source of proton release during intense exercise.

**Figure 11:**
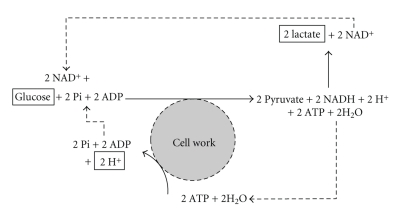
The changes and cycling of substrates, products, and protons during the glycolytic-lactate energy system. Adapted from Robergs et al. [45].

Figure 11 presents the net substrates and products of glycolysis, and how lactate production and the ATP hydrolysis supporting cell work are involved in the cycling of substrates and products as well as the net release of protons. Based on this depiction of the biochemistry, it is clear that lactate production contributes to the recycling of the protons released from glycolysis and that the protons released from ATP hydrolysis during cell work require removal from the cell or cytosol, or metabolic and structural buffering to prevent the development of metabolic acidosis. Once again, it is clear that lactate production is beneficial, not detrimental, to muscle contraction and metabolism during intense exercise. Nevertheless, despite the clear biochemical evidence against a lactic acid cause of metabolic acidosis, there remains strong inertia in science for continuing to use the simple lactic acid explanation of acidosis. The field of acid-based physiology is currently undergoing tremendous change and challenge to better explain and scientifically validate the true cause of metabolic acidosis [19, 36, 45, 47, 52].

## 6. Mitochondrial Respiration

The resynthesis of ATP by mitochondrial respiration occurs in mitochondria and involves the combustion of fuel in the presence of sufficient oxygen. The fuel can be obtained from sources within the muscle (free fatty acids and glycogen), and outside the muscle (blood free fatty acids [from adipose tissue], and blood glucose [from dietary ingestion or the liver]).

We will comment on the reactions involved in mitochondrial respiration structured by the source of substrate.

### 6.1. Carbohydrate Oxidation

**Figure 12:**
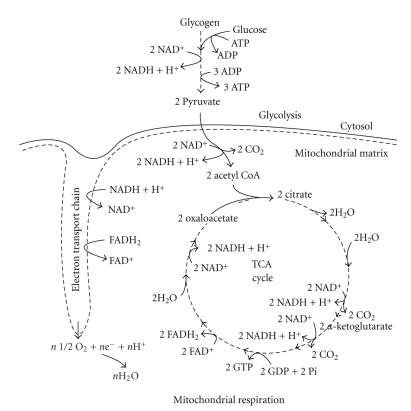
The biochemical connections between the cytosol and mitochondria of skeletal muscle for the complete oxidation of carbohydrate. Note that the TCA cycle as shown is not complete for all substrates and products. Also, the ATP yield from carbohydrate oxidation is greater for the substrate glycogen than glucose. For skeletal muscle, there are 37 molecules of ATP produced from one Glucose-6-phosphate derived from glycogen, assuming the presence of the glycerol-3-phosphate shuttle.

The connection between the mitochondria and glycolysis is complete when pyruvate and the electrons and protons from the glycolytic reduction of NAD^+^ to NADH are transferred into the mitochondria as substrates for mitochondrial respiration. Past scholars and researchers have referred to the involvement of glycolysis in the complete oxidation of carbohydrate as “aerobic glycolysis”, in contrast to the term “anaerobic glycolysis” when pyruvate is converted to lactate. We have problems with this terminology, as it dates back several decades to the pre-1980's when it was assumed that the extent of cell oxygenation was solely responsible for the complete oxidation of pyruvate via mitochondrial respiration. We now know this to be an incorrect assumption, for if exercise is intense enough lactate will always be produced regardless of normal oxygenation, or even hyperoxygenation such as with the breathing of pure oxygen. The labelling of glycolysis differently based on terms related to the presence or absence of oxygen is inconsistent with the biochemistry of glycolysis. Furthermore, the fact remains that the entire glycolytic pathway is oxygen independent or “anaerobic”. More biochemically correct alternate names would be “lactic glycolysis” versus “alactic glycolysis” for intense and steady state exercise conditions, respectively. Figure 12 summarizes the biochemical connections between the cytosol and mitochondria of skeletal muscle for the complete oxidation of carbohydrate.

### 6.2. Lipid Oxidation

Palmitate is the main form of fatty acid catabolized in skeletal muscle at rest and during muscle contraction. Palmitate is a 16 carbon fatty acid, and when in the cytosol of skeletal muscle must be activated by addition of coenzyme A prior to transport into mitochondria (4). This reaction is irreversible due to the energy change being so large. All fatty acids with 15 or more carbons require activation for transport into the mitochondria

(4) $$\begin{array}{cc} & \text{Fatty}\;\text{Acid}+\text{CoA}+\text{ATP}\\ & \;\;\overset{\text{acyl}\;\text{CoA}\;\text{synthetase}}{\to }\text{Fatty}\;\text{Acyl-CoA}+\text{PPi}+\text{AMP}. \end{array}$$

**Figure 13:**
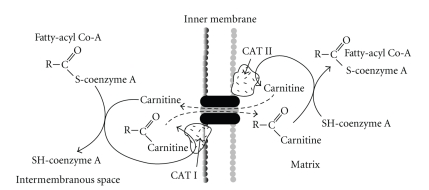
Transport of long chain (>15 carbons) fatty acyl CoA molecules into the mitochondria via the carnitine shuttle.

**Figure 14:**
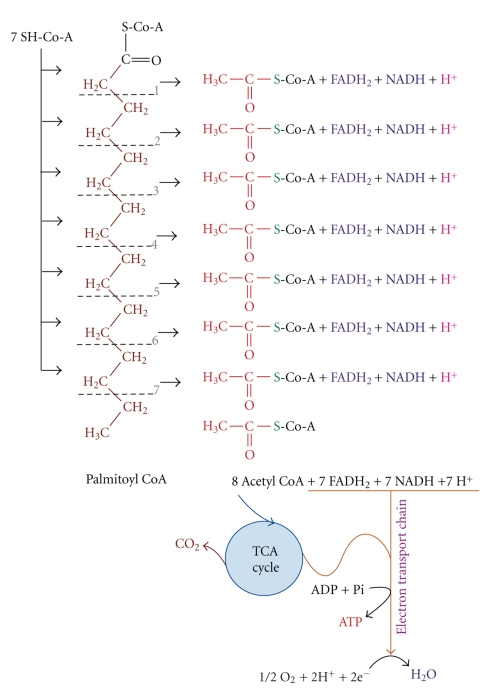
The sequential degrading of fatty acyl CoA molecules, as occurs in the *β*-oxidation pathway, within the mitochondria.

The inner mitochondrial membrane is impermeable to long chain fatty acids; therefore the fatty acyl CoA molecules are transported into the mitochondria via the carnitine shuttle, as shown in Figure 13. Once inside the mitochondria, saturated fatty acids, such as palmitate, are sequentially degraded two carbons at a time in the four reaction *β*-oxidation pathway, releasing acetyl CoA, 1 NADH, and 1 FADH per cycle (Figure 14).

**Figure 15:**
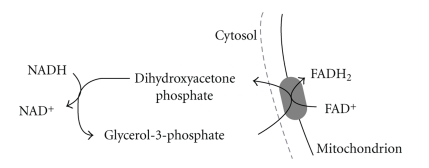
Glycerol-3-phosphate shuttle. The reduction of FAD^+^ within the mitochondria occurs within the TCA cycle, where the enzyme glycerol-3-phosphate dehydrogenase is linked within succinate dehydrogenase (TCA cycle) within complex II of the electron transport chain.

Note that the acetyl CoA is produced from *β* oxidation then enters the TCA cycle like that for the oxidation of acetyl CoA derived from pyruvate oxidation. As such, the products of fatty acid oxidation per acetyl CoA are identical. Differences between fatty acid oxidation and carbohydrate oxidation must therefore occur prior to and during the production of acetyl CoA. When a comparison between the products of glucose, glycogen, and palmitate oxidation to 8 acetyl CoA molecules is made, carbohydrate oxidation yields a higher proportion of NADH to FADH_2_, more CO_2_, and a greater ATP yield, even when accounting for the less ATP efficient glycerol-3-phosphate shuttle for electron transfer from glycolysis (NADH + H^+^) into the mitochondria. This occurs through the glycerol-3-phosphate shuttle (Figure 15), which is prominent in muscle and so enables the muscle to maintain a very high rate of oxidative phosphorylation. When cytosolic NADH transported by the glycerol-3-phosphate shuttle is oxidised by the respiratory chain 1.5 ATP is produced, rather than 2.5 ATP. This is due to FAD, not NAD^+^, being the electron acceptor. FAD allows NADH to be transported into the mitochondria against a concentration gradient; this occurs at a cost of 1ATP molecule per 2 electrons [17]. The higher ATP yield means that for a given rate of ATP regeneration there would be less demand for oxygen consumption. In addition, such ATP regeneration occurs with a higher CO_2_ production, explaining the lower respiratory exchange ratio (RER) during exercise for a greater reliance on lipid than carbohydrate oxidation.

### 6.3. Amino Acid Oxidation

Muscle has an available supply of amino acids for use in catabolism, and these comprise what is known as the free amino acid pool. However, continued muscle contraction, especially when carbohydrate supply and/or provision is inadequate, requires protein catabolism to sustain free amino acids. Thus, prolonged exercise in times of poor carbohydrate nutrition increases protein breakdown and amino acid oxidation. Intense exercise also increases amino acid oxidation, but involves negligible protein catabolism due to the short-term nature of intense exercise.

## 7. Conclusions

The interaction and relative contribution of the 3 energy systems during incremental exercise and periods of maximal exhaustive exercise are of considerable theoretical and practical interest. The energy systems respond differently in relation to the high, often sustained and usually diverse energy demands placed on them during daily and sporting activities. Analysis of the current literature suggests that virtually all physical activities derive some energy from each of the 3 energy-supplying processes. There is no doubt that each system is best suited to providing energy for a different type of event or activity, yet this does not imply exclusivity. Similarly, the energy systems contribute sequentially but in an overlapping fashion to the energy demands of exercise.

The anaerobic (nonmitochondrial) system is capable of responding immediately to the energy demands of exercise and is able to support extremely high muscle force application and power outputs. Unfortunately the anaerobic system is limited in its capacity, such that either a cessation of work or a reduction in power output to a level that can be met by aerobic metabolism is seen during extended periods of intense exercise. The aerobic energy system responds surprisingly quickly to the demands of intense exercise, yet due to a relatively low rate of ATP turnover, is incapable of meeting the energy demands at the beginning of exercise, irrespective of the exercise intensity, or intense exercise. Nevertheless, it now seems evident that the aerobic system plays a significant role in determining performance during high-intensity exercise, with a maximal exercise effort of 75 seconds deriving approximately equal energy from the aerobic and anaerobic energy systems.
